# Observance de l’hygiène des mains dans les hôpitaux généraux de référence de la ville de Kisangani en République Démocratique du Congo

**DOI:** 10.11604/pamj.2020.35.57.18500

**Published:** 2020-02-26

**Authors:** Eugène Basandja Longembe, Panda Lukongo Kitronza

**Affiliations:** 1 Faculté de Médecine et de Pharmacie, Université de Kisangani, Kisangani, République Démocratique du Congo; 2 Ecole de Santé Publique, Faculté de Médecine, Université de Liège, Liège, Belgique

**Keywords:** Observance, hygiène des mains, Hôpital Général de Référence de Kisangani, Compliance, hand-hygiene practices, GRH Kisangani

## Abstract

**Introduction:**

la présente étude avait pour objectif d’évaluer le niveau de l’observance de l’hygiène des mains par les professionnels de santé des Hôpitaux Généraux de Référence (HGR) de la ville de Kisangani et d’identifier les facteurs déterminants.

**Méthodes:**

une étude transversale est menée dans les services de maternité, chirurgie, pédiatrie et urgences des quatre HGR de la ville de Kisangani pendant la période du 13 au 20 juin 2018. Un questionnaire auto-administré a été remis à 120 professionnels de santé recrutés parmi les médecins, infirmiers, techniciens de laboratoire et garçons et filles de salle pour évaluer leur niveau de connaissance et une grille d’observation de l’hygiène des mains utilisée sur 44 prestataires pour 1920 opportunités.

**Résultats:**

le taux d’observance global de l’hygiène des mains est de 39% [IC95 0,37; 0,41]; la friction avec la solution hydro alcoolique est beaucoup moins fréquente (5%); les techniciens de surface et les médecins ont présenté des taux d’observance plus élevés respectivement 49% et 44% que les infirmiers (33%). Environ un tiers de professionnels connaissent les indications de l’hygiène des mains selon l’OMS; 37% de prestataires ont déclaré avoir suivi une formation en cours d’emploi sur l’hygiène des mains et 36% connaissent l’importance de l’hygiène des mains en milieu de soin. La différence de connaissance n’est pas significative entre les catégories professionnelles étudiées (p > 0,05).

**Conclusion:**

cette étude nous a permis à travers les résultats obtenus de conclure que le niveau d’observance des précautions standards en hygiène des mains par les professionnels de santé reste insuffisant. Il s’avère donc nécessaire de renforcer l’observance de l’hygiène des mains par les programmes de formation et de sensibilisation des professionnels de santé, l’approvisionnement en produits d’hygiène et la prise de conscience des prestataires de soins.

## Abstract (en)

**Introduction:**

the purpose of this study was to assess the compliance with hand-hygiene practice of health-care workers in the General Reference Hospitals (GRH) of the city of Kisangani and to identify factors contributing to it.

**Methods:**

we conducted a cross-sectional study in the Maternity, Surgery, Pediatric and Emergency Departments at four GRH of the city of Kisangani over the period 13^th^-20^th^ June 2018. One hundred and twenty professionals recruited from among doctors, nurses, laboratory technicians and attendants were asked to complete a self-administered questionnaire to assess their level of knowledge and a grid indicating the compliance with hand-hygiene practice in 44 health professionals (1920 opportunities).

**Results:**

the rate of overall compliance with hand-hygiene practice was 39% [CI95 0.37; 0.41]; friction with hydroalcoholic solution was much less frequent (5%); cleaners and physicians had higher compliance rates (49% and 44% respectively) than nurses (33%). Approximately one third of professionals were aware of the indications for hand-hygiene according to the WHO; 37% of health professionals declared that they had followed a on-the-job training on hand-hygiene and 36% knew the importance of hand-hygiene in the healthcare environment. The gap in knowledge was not significant between the occupational categories studied (p >0.05).

**Conclusion:**

this study and the results obtained from it allowed us to conclude that the level of compliance to precautions standards including hand hygiene by healthcare professionals is insufficient. It is therefore necessary to strengthen the compliance with hand-hygiene practices through training and awareness programs for healthcare professionals, the supply of hygiene products and the awareness of healthcare providers.

## Introduction

Les infections associées aux soins sont fréquentes et constituent un problème majeur de santé publique [1]. Elles sont fréquemment les résultats de soins non-sécurisés [2]. Ces infections constituent une menace sérieuse dont l’impact économique pour les patients et les systèmes de santé dans le monde entier est significatif [1]. Au-delà des conséquences physiques et morales subies par les patients et leurs familles, les Infections Associées aux Soins (IAS) ont des conséquences graves. Elles entrainent une prolongation de séjour d’hospitalisation [3, 4], des frais excédentaires [5, 6], des résistances élevées des micro-organismes aux antibiotiques et des taux de mortalité élevés [7–10]. Elles représentent une charge financière élevée pour les systèmes de santé et engagent des ressources qui pourraient être allouées au financement de mesures préventives ou d’autres actions prioritaires [5, 6]. Les IAS sévissent partout dans le monde et affectent des centaines de millions de patients dans les pays développés comme dans les pays en développement. Dans les pays développés, les Infections Associées aux Soins (IAS) touchent 5 à 10% des patients admis dans les établissements de soins intensifs. Dans les pays en développement, le risque est 2 à 20 fois plus élevé et le pourcentage de patients affectés est parfois supérieur à 25% [2, 11–13]. Dans les pays en développement, ces infections étaient estimées entre 5 à 15% des patients hospitalisés et peuvent affecter entre 9 et 37% des patients admis dans les unités de soins intensifs [11, 13, 14]. En Afrique, les taux de prévalence d’infections contractées au cours des soins varient entre 14,8% à 19,1% [13, 15]. Au Maroc, environ 10% des patients hospitalisés contractent une infection nosocomiale. Cependant, 70% de ces infections sont imputables au manuportage [16]. Les mains de prestataires de soins constituent la principale source de transmission d’infection en milieu de soins (flore résidente et flore transitoire). L’hygiène des mains reste la mesure la plus importante et la plus efficace des précautions générales dans la prévention des infections liées aux soins et la dissémination de micro-organismes multi-résistants, la prévention du transfert de micro-organismes entre patients, soignants et environnement hospitalier [16–18]. L’hygiène des mains en milieu hospitalier devient donc une priorité afin de réduire les infections nosocomiales (IN), aussi appelées infections associées aux soins de santé (IASS). Plusieurs études montrent que l’amélioration de 30% de l’observance de l’hygiène des mains est associée à une diminution de 41% de la prévalence des infections [19, 20]. Malgré les multiples initiatives de promotion et de formation, les données de la littérature issues de pays développés font mention d’observance de l’hygiène des mains inférieure à 60% chez les professionnels de santé [21, 22]. La connaissance des professionnels de santé sur l’hygiène des mains en milieu de soin est insuffisante, de surcroit, les pratiques restent défectueuses. Malgré le rôle de modèle des médecins vis-à-vis des autres travailleurs, leur observance reste très faible, environ moins 20% par rapport aux infirmiers [23, 24]. Les facteurs de faible observance mis en exergue lors d’une étude menée au Maroc étaient l’insuffisance de formation en cours d’emploi, l’insuffisance de produits de l’hygiène des mains et la charge de travail excessive des prestataires de soins [25]. Les campagnes de sensibilisation des prestataires de soins et de promotion de l’hygiène des mains organisées régulièrement dans région du monde, est une stratégie probante pour améliorer l’observance des règles de l’hygiène des mains à court et à long termes [23, 25–27]. Les informations sur le risque infectieux et la perception des professionnels de santé sur ce risque sont peu documentées dans la littérature africaine [20, 28]. La démarche qualité de soins et la sécurité de patients est faiblement appliquée et elle est liée entre autres à la faible implication et à l’insuffisance de politiques institutionnelles des hôpitaux en la matière [15, 23, 29]. La compréhension des mécanismes de transmission des micro-organismes pendant les soins aux patients est certainement un élément moteur dans l’amélioration de l’observance de l’hygiène des mains [16, 30]. Malgré les multiples initiatives de promotion et de formation, les données de la littérature issues de pays développés font mention d’un taux d’observance à l’hygiène des mains inférieur à 60% chez les professionnels de la santé [17, 31, 32]. Ainsi, l’observance des règles d’hygiène des mains par professionnels de la santé laisse encore à désirer de par le monde et surtout dans les pays en développement [2, 11, 13, 16, 33–35]. Les hôpitaux de la RDC et ceux de la ville de Kisangani en particulier ne déroge pas à cette problématique. Pour être efficace dans le contrôle des IAS par le manuportage dans les établissements de soins de la ville de Kisangani, il est important de disposer des informations valides sur les connaissances, les attitudes et les pratiques des professionnels de santé sur l’observance de l’hygiène des mains en milieu de soins. Ainsi, cette étude est menée pour évaluer l’observance de l’hygiène des mains par les professionnels de santé des Hôpitaux Généraux de Référence (HGR) de la ville de Kisangani et d’identifier les facteurs déterminants et renforcer les moyens de préventions.

## Méthodes

### Type d’étude et échantillonnage

Nous avons mené une étude descriptive transversale exhaustive pour la période du 13 au 20 juin 2018 dans quatre hôpitaux généraux de référence de la ville de Kisangani à savoir HGR Mangobo, Lubunga, Kabondo et Tshopo. Les enquêtes ont été réalisées dans 4 services de chaque institution hospitalière (pédiatrie, maternité, urgences et chirurgie) auprès de toutes les catégories professionnelles présentes dans les services ciblés le jour de l’enquête. Les professionnels de santé éligibles comprenaient les médecins, les infirmiers, les techniciens de laboratoire et les techniciens de surface (garçons et filles de salle) prestant dans les différents services ciblés au sein de chaque HGR. L’exigence principale de la méthodologie de l’observation de l’hygiène des mains telle que définie par l’OMS est la représentativité des professionnels observés en termes de catégorie professionnelle et d’environnement de soins [23]. Idéalement, la taille de l’échantillon est calculée au moment de la planification de l’observation des pratiques de l’hygiène des mains. Néanmoins, les estimations de taille d’échantillon indiquent que 200 opportunités par période d’observation et par unité (d’observation/d’analyse/de comparaison), tels que l’unité de soins, le service, la catégorie professionnelle, etc., sont nécessaires pour une comparaison fiable des résultats [23]. Ainsi, chaque HGR était considéré comme une strate de 30 professionnels de santé, toutes catégories professionnelles confondues, répartis dans les 4 services ciblés. Les professionnels de santé présents dans le service le jour et au moment de l’étude étaient enquêtés jusqu’à atteindre la taille de l’échantillon attendue.

### Technique de collecte des données

Les informations ont été recueillies aux moyens d’un questionnaire d’enquête auto-administré remis aux professionnels de santé de toutes les catégories professionnelles ciblées, présent dans le service au moment de l’étude. Les données récoltées ont été en rapport avec le niveau de connaissance d’une part et l’attitude des professionnels de santé, d’autre part. En plus du questionnaire, une grille d’observation pour les aspects pratiques et l’observation de l’hygiène des mains selon les 5 indications de l’OMS, a été utilisée [36]. Quatre enquêteurs n’appartenant pas aux HGR ciblés ont été formés et déployés dans les services ciblés des HGR pendant un jour pour évaluer l’observance de l’hygiène des mains. Ces enquêteurs ont observé les prestataires pendant 15 à 30 minutes (20 minutes en moyenne) pour compter les opportunités en rapport avec les 5 indications de l’hygiène des mains et enregistré sur la grille d’évaluation le comportement des prestataires comme suit: lavage des mains avec de l’eau et du savon, l’usage de solution hydro alcoolique (SHA) ou rien. Le nombre d’opportunités prévu par l’OMS est de 200 [23]. Plusieurs sessions d’observation de 20 minutes en moyenne ont été organisées par service et par catégorie professionnelle jusqu’à atteindre le nombre voulu.

### Analyse des données

Les données ainsi collectées étaient encodées et analysées à l’aide de logiciel SPSS 20. Les statistiques descriptives ont porté sur la mesure de fréquence et la proportion. Pour les inférences statistiques, nous avons utilisé pour certains tableaux le test de chi carré de Pearson et le P value a été obtenu en utilisant les tables qui donnent les valeurs de la distribution théorique que suis le test calculé; et pour d’autres tableaux, nous avons utilisé l’intervalle de confiance au seuil de signification de 0,05 pour comparer les proportions estimées. L’IC_95_ a été calculé suivant la formule:

$$\text{p}\pm \text{Z}\sqrt{pq/d}$$

### Variables d’intérêt

Les variables d’intérêt ciblées dans cette étude sont: 1) caractéristiques des participants: âges, sexe, catégories professionnelles; 2) connaissance des participants sur la définition correcte de l’hygiène des mains, son importance, les différentes techniques et les indications de l’OMS; 3) taux d’observance de l’hygiène des mains par service et par catégories professionnelles. Le taux d’observance a été calculé globalement et par service et par catégorie professionnelle comme suit:

$$\frac{\Sigma pratiques\;del’hygiène\;de\;mains\;(lavage+friction\;SHA)\;observé}{nombre\;total\;d’opportunitès}$$

### Considérations éthiques

Tout participant à cette étude était informé de l’objectif poursuivi et de la méthodologie retenue pour la collecte de ses informations. Un consentement verbal a été obtenu et des garanties que les données recueillies seront analysées dans l’anonymat ont été données. Les personnes ciblées n’ayant pas consenti n’ont pas été interviewées (4 non réponse).

## Résultats

Au total 120 professionnels sur 124 contactés soit 97% ont répondu au questionnaire pour l’évaluation de connaissance et la formation en cours d’emploi dans le domaine d’hygiène des mains en milieu de soin et 44 professionnels ont été observés pour un total de 1920 opportunités en rapport avec l’observance de l’hygiène des mains. La majorité des enquêtés était des infirmiers (65%) avec prédominance de sexe féminin (59,7%) avec l’âge moyen de 44,3 ans ± 12,4 (Tableau 1). Moins de la moitié des professionnels de santé (46%) ont une bonne connaissance de la définition de l’hygiène des mains, la fréquence la plus élevée (70%) est observée parmi les médecins mais de manière non significative par rapport aux autres catégories professionnelles (p > 0,05). La même observation est faite pour les professionnels de santé qui connaissent l’importance de la pratique de l’hygiène des mains (37%) et ceux déclarant avoir bénéficié d’une formation en cours d’emploi sur l’hygiène des mains (36%) (p>0,05) (Tableau 2). La friction des mains à l’aide de solution hydro alcoolique est une technique moins connue (6%), cette connaissance est différente de celle de lavage des mains avec de l’eau et du savon (40% IC_95_ 31-49). Les techniciens de laboratoire (67%) et les médecins (65%) ont une connaissance moyenne élevée de deux techniques de l’hygiène des mains (utilisation de l’eau et du savon et friction hydro alcoolique) par rapport aux autres catégories professionnelles et à la moyenne observée (44%). La connaissance de chacune de 5 indications de l’hygiène des mains est en moyenne faible (<50%) et pas différente (IC_95_). Environ 1/4 (24%) des professionnels de santé seulement connaissent les 5 indications de l’hygiène des mains selon l’OMS (Tableau 2). Le taux global d’observance de l’hygiène des mains est de 39% (IC95 37; 41) avec une prédominance de lavage avec de l’eau et du savon (34%) sur la friction des mains à l’aide de solution hydro alcoolique (5%), le gold standard (Tableau 3).

**Tableau 1:** Caractéristiques de l’échantillon

Caractéristiques	Professionnel de santé répondant au questionnaire (n=120)	Professionnels de santé observés (n=44)
Sexe F, (%)	68 (59,7)	23 (52,3)
Age moyen, ET	44,3 (±12,4)	40,1 (± 11)
**Profession n, %**		
Infirmiers	78 (65)	24 (54,5)
Médecins	20 916,7)	12 (27,3)
Filles et Garçons de salle	16 (13,3)	8 (18,2)
Techniciens de laboratoire	6 (5)	-

**Tableau 2:** Connaissances générales des professionnels de santé et formation en cours d’emploi sur l’hygiène des mains

Rubriques	Médecin (n=20)	Infirmier (n=78)	Technicien labo (n=6)	Techn.Surf^a^. (n=16)	Ensemble (n=120)
Connaissance de la définition correcte de l’hygiène des mains					
OUI (*Khi deux=6,61; p=0,085)*	14 (70)	34 (44)	2 (33)	5 (31)	55 (46)
Formation en cours d’emploi sur l’hygiène des mains suivie					
Oui (*Khi deux=1,596; p=0,660)*	8 (40)	25 (32)	3 (50)	7 (44)	43 (36)
Connaissance de l’importance de l’hygiène des mains en milieu de soins comme moyen de prévention des IAS					
OUI (*Khi deux=4,31; p=0,230)*	11 (55)	26 (33)	1 (17)	6 (38)	44 (37)
**Connaissance des différentes techniques de l’hygiène des mains (IC_95_)**					
Ne sait pas	0 (0)	7 (9)	0 (0)	5 (31)	12 (10) [5-15]
Eau avec savon	6 (30)	32 (41)	1 (17)	9 (56)	48 (40) [31-49]
Solution hydro alcoolique	1 (5)	5 (6)	1 (17)	0 (0)	7 (6) [2-10]
Eau et SHA	13 (65)	34 (44)	4 (67)	2 (13)	53 (44) [35-53]
**Connaissance des indications de l’hygiène des mains selon l’OMS (IC_95_)**					
Avant tout contact patient	9 (45)	27 (35)	1 (17)	3 (19)	40 (33) [25-41]
Avant tout acte septique et propre	7 (37)	21 (27)	1 (17)	3 (19)	32 (27) [19-35]
Après risque liquide biologique	9 (45)	22 (28)	0 (0)	2 (13)	33 (28) [20-36]
Après contact patient	8 (40)	23 (29)	1 (17)	2 (13)	34 (28) [20-36]
Après contact environnement patient	7 (37)	19 (24)	0 (0)	1 (6)	27 (23) [15-30]
Connaissance de 5 indications	7 (37)	21 (27)	0 (0)	1 (6)	29 (24) [16-32]

a Techniciens de surface [Garçon de Salle + Fille de Salle)

**Tableau 3:** Résultat de l’observance de l’hygiène des mains

Indications	Nb opportunités	Nb observation	Taux d’observance
**Observance globale [alcool+ savon/total)**	1920	742	39% [IC_95_ 37-41]
**Observance par lavage avec de l’eau et savon**	1920	649	34% [IC_95_ 32-36]
**Observance par l’application de Solution Hydro Alcoolique**	1920	93	5% [IC_95_ 4-6]
**Observance par catégories professionnelles**			
Médecin	567	249	44% [IC_95_ 40-48]
Infirmiers	1028	335	33% [IC_95_ 30-36]
Techniciens de surface	325	158	49% [IC_95_ 44-54]
**Observance par service**			
Chirurgie	480	177	37% [IC_95_ 33-41]
Maternité	480	196	41% [IC_95_ 37-45]
Pédiatrie	480	178	37% [IC_95_ 33-41]
Urgences et réanimation	480	191	40% [IC_95_ 36-44]

Le taux d’observance est élevé chez les techniciens de surface (garçons et filles de salle) et les médecins respectivement 47% (IC_95_ 44; 54) et 44% (IC_95_ 40; 48); il est significativement différent par rapport aux infirmiers 33% (IC_95_ 30; 36). Il n’y a pas de différence d’observance en rapport avec les services de maternité (Tableau 3).

## Discussion

Notre étude est basée sur une enquête sur l’observance, chez les soignants, de l’hygiène des mains dans les HGR de la ville de Kisangani. Les résultats ont montré une mauvaise application de l’observance de l’hygiène en rapport avec les précautions standards. Le taux d’observance global de l’hygiène des mains trouvé dans cette étude est de 39% (IC95 0,37; 0,41) dans les HGR de la ville de Kisangani. Ce taux est plus faible que ceux rapportés en Belgique avant la première campagne de sensibilisation et de promotion de l’hygiène des mains de 2014-2015 (69,1%) [24] et à Bamako lors de la première phase (52,05%) [29]. Suchitra *et al.* ont trouvé aussi une compliance de l’hygiène des mains supérieure à la nôtre (63,3%) avec un taux plus élevé parmi les techniciens de surface (76,7%) [27]. Par contre, le taux d’observance beaucoup plus faible a été observé au cours de l’étude menée par Mathai *et al.* dans l’unité de soins intensifs (26%) [37] et Sakihama *et al.* qui a trouvé l’observance générale à 19% (IC_95_ 18%; 20%) avec une prédominance des infirmiers (23%) par rapport aux médecins (15%) [38].

Plusieurs facteurs ont été identifiés en association à la faible observance de l’hygiène des mains aussi bien dans la littérature que dans cette étude. L’insuffisance des prestataires formés en cours d’emploi sur l’hygiène des mains (36%) (IC_95_ 0,35; 0,37) serait un des facteurs associés tel que reconnu dans la littérature. De surcroit il est observé une proportion faible des prestataires qui connaissent la définition correcte de l’hygiène des mains en milieu de soins (46%) et de son importance (36%), la connaissance de 5 indications de l’hygiène des mains selon l’OMS (environ 1/3). La différence de connaissance n’est pas significative entre les différentes catégories professionnelles (la proportion de prestataires formés trouvée dans cette étude est faible par rapport à celle rapportée par Derraji au Maroc (60%) [25]. L’inexistence des politiques institutionnelles de promotion et de suivi de l’application de l’hygiène des mains, l’insuffisance de ressources en appui au fonctionnement des établissements de soins, le manque d’infrastructures adéquates constituent des barrières importantes et seraient à l’origine de faible taux d’observance, comme le suggère l’étude faite par Ataiyero [28].

Les actions de sensibilisation et de formation ont été identifiées par Benali Beghdadli *et al.* comme des actions prioritaires à mettre en œuvre pour améliorer l’observance de l’hygiène des mains [39]. Toutes ces évidences restent valables pour notre milieu d’étude. Il est trouvé dans cette étude que l’usage de solution hydro alcoolique était très faible (5%) et pratiqué essentiellement par les médecins. Cette faiblesse a été également observée au Maroc (19%) [25]. Il va sans dire que la promotion de l’hygiène des mains n’est possible que si les ressources informationnelles et matérielles sont disponibles [28, 30]. Au sujet de catégories professionnelles, contrairement à ce qui a été trouvé dans la littérature, la bonne observance chez les infirmiers par rapport aux médecins avec un écart d’environ 20% [18, 24, 25, 38, 40], l’observance était élevée chez les médecins (44%) (IC_95_ 40-48) que chez les infirmiers (33%). Nos résultats corroborent avec ceux trouvés au Ghana en 2009 où le taux d’observance avant et après contact patient était élevé chez les médecins par rapport aux infirmiers respectivement de 15,4% et 38,5% (médecins) contre 14,1% et 9,9% (infirmiers) [40] et l’Inde en 2011 où le taux de non-conformité était plus élevé chez les infirmiers que les médecins [41]. La qualification professionnelle constitue un avantage pour la mise en œuvre efficace des activités de soins et de service. Ceci est aussi nécessaire pour ce qui concerne l’observance de l’hygiène pour la prévention des infections associées aux soins [40]. Des preuves indirectes sont aussi apportées par des investigations d’épidémies qui montrent d’une part que l’adhésion à des mesures de prévention comme l’hygiène des mains entraîne une diminution du taux d’attaque des pathogènes nosocomiaux [42, 43], mais aussi qu’il y a souvent un lien entre apparition d’épidémies, augmentation de la charge de travail, pénurie de personnel et mauvaise observance de l’hygiène des mains [40, 44].

Cette situation dans notre étude se justifierait par le fait que, chaque médecin dispose d’un flacon de solution hydro alcoolique et que les infirmiers, par contre, utilisent dans la quasi-totalité de cas, de l’eau et du savon pour pratiquer l’hygiène des mains. Le manque de dispositif de lavage des mains au chevet de malades influe négativement sur la pratique de l’hygiène des mains lors de l’administration de soins. La fourniture de désinfectants à base d’alcool au chevet des malades a été également identifiée comme une des solutions pour améliorer l’observance de l’hygiène des mains [42]. L’introduction de l’usage de solution hydro alcoolique dans les consommables des établissements de soins est connue comme une des stratégies multimodales efficace pour améliorer l’observance de l’hygiène des mains. Cette politique institutionnelle n’est pas encore à jour dans les hôpitaux généraux de la ville de Kisangani [1, 35, 45–47]. Dans d’autres études, il a été observé que les indications de l’hygiène des mains les plus respectées étaient « après contact patient et après exposition au risque de liquide biologique » que « avant contact patient » confirmant ainsi l’hypothèse selon laquelle le soignant a tendance à se protéger d’abord [1, 18, 39, 46]. Cette théorie pourrait justifier l’observance plus élevée notée dans cette étude parmi les techniciens de surface. Dans le cas de notre étude, l’absence de différence serait due au fait que les sessions d’observation en chirurgie étaient plus réalisées dans le service d’hospitalisation de chirurgie que dans le bloc opératoire et dans la salle de soins post partum que dans la salle d’accouchement où l’accès des enquêteurs était limité. Garçons et filles de salle à 49%, les taches essentielles exécutées par cette catégorie professionnelle sont en rapport avec l’environnement des patients et les matériels de soins souillés contre lesquelles ils se protègent.

L’observance est quasi la même entre les différents services étudiés, environ 38%. Dans la littérature environ 38%. Dans la littérature, il est reconnu un taux d’observance plus élevée en chirurgie et à la maternité à la suite de la fréquence élevée des actes invasifs et de l’exposition aux liquides biologiques. Cela rejoint les observations faites à Bamako il est reconnu un taux d’observance plus élevée en chirurgie et à la maternité à la suite de la fréquence élevée des actes invasifs et de l’exposition aux liquides biologiques. Cela rejoint les observations faites à Bamako [29].

### Limites

Le choix de la méthodologie peut constituer une des principales limites de cette étude. En effet, les auto-déclarations permettent d’évaluer les connaissances plus que les pratiques professionnelles. En plus, il est à noter que l’objectivité apparente des réponses à un auto-questionnaire et l’auto-évaluation comme démarche peut entraîner un biais de réponses [18]. Il sied de noter également la limite inhérente au caractère d´une étude uni centrique, avec une taille d´échantillon modeste. Enfin, l’étude peut être influée par un biais de sélection, liée à la taille de l’échantillon et dont l’effet sur les estimations est difficilement appréciable. Ce biais peut éventuellement remettre en cause l´inférence de nos résultats, compte tenu notamment de la situation sociogéographique particulière à chaque institution hospitalière. Toutefois, les biais pouvant provenir des déclarations erronées du fait de nos variables dichotomiques ont été minimisés par la traduction de notre questionnaire en langue locale. Le biais de mémoire qui affecterait les réponses à notre questionnaire a été limité par la formulation des questions en rapport avec les problèmes de santé, à travers un temps de référence très court. Quant au biais d’interview provenant de la formulation des réponses données, il a été limité par la formation des enquêteurs.

## Conclusion

En dépit des limites susmentionnées, il se dégage de cette étude que le taux d’observance de l’hygiène des mains dans les HGR de la ville de Kisangani est très faible indistinctement de services et de catégories professionnelles. Ceci ne permet pas d’interrompre la chaine de transmission manuportée des infections en milieu de soins. La pratique de l’hygiène des mains par les professionnels soignants est une approche efficace de réduction de ces infections. Elle est favorisée par la formation, la disponibilité des ressources favorables à la pratique ainsi que l’information sur les conditions d’utilisation des solutions hydro alcooliques. Il va de soi qu’une étude de prévalence des IAS dans ces établissements de soins montrera des taux plus élevés de ces infections. La mise en place des politiques nationale et locale de formation et de sensibilisation permanente des professionnels de santé en cours d’emploi et d’approvisionnement (production locale des SHA, achat, subvention) des produits d’hygiène des mains doit être envisagée afin de renforcer ces pratiques qui ont prouvé leur efficacité dans les programmes et les stratégies de prévention du risque infectieux. Une prise de conscience des médecins et infirmiers est nécessaire au regard du rôle qu’ils ont à jouer en tant que garants de l’amélioration de l’état de santé de la population à travers les soins sécurisés requis par l’OMS. Notons que les résultats de notre étude pourraient simplement suggérer l’état de lieu, mettre en exergue la problématique en matière d’observance de l’hygiène des mains et de mettre en place certaines mesures préliminaires dans les HGR de la ville de Kisangani. Ils sont donc à confronter avec d’autres études multicentriques méthodologiquement robustes en vue d’une action politique appropriée, efficace et efficiente.

### Etat des connaissances actuelles sur le sujet

- En Afrique et dans certains pays en développement, le taux le plus élevé de prévalence de ces infections est estimé à 25,0%;
- L’observance de l’hygiène des mains est toujours faible lors des premières sessions d’évaluation;
- Les raisons de la faible pratique de l’hygiène des mains n’ont pas été clairement définies dans les pays en développement, probablement en raison du nombre limité d’observations et d’études sur l’hygiène des mains.

### Contribution de notre étude à la connaissance

- Cette étude a mis en évidence le faible taux d’observance de l’hygiène des mains dans les HGR de la ville de Kisangani, indistinctement de services et de catégories professionnelles;
- L’analyse a permis d’identifier les facteurs clés d’amélioration: l’ergonomie des chambres dont l’infrastructure; la disponibilité des ressources favorables à la pratique ainsi que la formation et l’information sur le port de gants et sur les conditions d’utilisation des solutions hydro alcooliques; la formation pédagogique doit être optimisée et restructurée dans l’objectif unique de pérenniser la prise de conscience et la compliance des personnels soignants pour les soins sécurisés requis par l’OMS;
- L’étude constitue donc un plaidoyer pour l’élaboration des politiques institutionnelles incitatives (hôpitaux) de sensibilisation, dotation des lignes budgétaires allouées aux activités de l’hygiène des mains (achat consommable et formation) pour le soutien de la promotion de l’hygiène des mains en milieu de soins.

## Conflits d’intérêts

Les auteurs ne déclarent aucun conflit d’intérêts.
